# The role of 3D technology in the practical education of congenital coarctation and its treatment—a feasibility pilot study

**DOI:** 10.1186/s12909-024-05293-6

**Published:** 2024-03-29

**Authors:** Imre J. Barabas, Daniel Vegh, Olivia Bottlik, Patrik Kreuter, Istvan Hartyanszky, Bela Merkely, Daniel Palkovics

**Affiliations:** 1 SE3D Center, Semmelweis University, Budapest, Hungary; 2 Department of Prosthodontics, Semmelweis University, Budapest, Hungary; 3 Heart and Vascular Center, Semmelweis University, Budapest, Hungary; 4 Department of Periodontology, Semmelweis University, Budapest, Hungary

**Keywords:** 3D printing, Education, Practical training, Synthetic bench model, Coarctation of the aorta, HOST

## Abstract

**Background:**

Coarctation of the aorta (CoA) is a congenital disease with an incidence of 4 out of 10,000 live births, therefore proper education of its treatment is essential. Understanding the disease and the wide array of treatment options is often difficult. Additive manufacturing technology can be used to produce 3D printed hands-on surgical training tools (HOSTT), which can be used for the education and practical training of CoA. This study aimed to investigate the effectiveness of a 3D printable HOSTT for the simulation of coarctation surgery, and it’ possible role in practical education.

**Methods:**

Participants were medical students of Semmelweis University between the second and sixth academic year. A virtual 3D model of an aorta with CoA was generated from a computed tomography angiography scan. Each participant received a 3D-printed aorta phantom and performed either one of four surgical treatment modalities. The simulated surgeries included end-to-end anastomosis, end-to-side anastomosis, prosthetic patch, and subclavian flap aortoplasty. Participants provided feedback, evaluating their understanding of the disease and its treatment by the four surgical reconstruction modalities on a seven-point Likert scale before and after the sessions.

**Results:**

21 medical students participated in this study. Participants’ average rating of their understanding of CoA disease and it treatment options before practical training was 4.62 ± 1.07. After training, their average rating increased to 6.19 ± 1.08, showing statistically significant difference.

**Conclusions:**

Within this study’s limitations, the applied HOSTT, manufactured using 3D printing, was effective for the practical training of CoA’s surgical treatment methods for medical students.

## Introduction

Advancements in 3D technologies have expanded their range in various surgical application. In clinical practice, 3D technologies can be used prior to surgical interventions (e.g., diagnosis, treatment planning, and treatment simulation) or intraoperatively (e.g., fabrication of anatomic models for intraoperative use, real-time image-guided therapy, and additive manufacturing of individualized surgical guides). In addition to clinical use, 3D technology plays an increasingly prominent role in graduate and postgraduate education and training [1–3].

Medical treatment simulation can either use reality-based or virtual methods. Reality-based methods include (i) human cadaver models, (ii) animal models, (iii) hands-on surgical training tools (HOSTTs), and (iv) full immersion models. Virtual methods use virtual- or augmented reality environments for simulation [4]. Compared to virtual methods, reality-based applications have the advantage of providing proper haptic feedback. Due to the limited accessibility of virtual models or 3D technology, traditional cadavers and animal models can be considered the gold standard. However, the application of such models is limited by ethical and financial constraints [5].

The development of medical imaging modalities, computer-aided design (CAD) software, and additive manufacturing (3D printing) technologies have enabled the production of realistic, life-like anatomical models for surgical simulation. HOSTTs are far easier to fabricate than full immersion models, where the entire operating theatre is simulated including the use of surgical dummies, tools and lights. HOSTTs are designed to replicate an organ with a known pathological condition, which is used to perform specific critical steps of a procedure [6].

Coarctation of the aorta (CoA) accounts for 5–8% of all congenital heart disease cases, with an incidence of four in every 10,000 live births [7, 8]. CoA can be defined as the stenosis of the thoracic aorta, most commonly located near the entrance of the ductus arteriosus [9]. Multiple surgical solutions to treat CoA were described between 1940 and 1970: (i) end-to-end anastomosis [10], (ii) end-to-side anastomosis [11], (iii) prosthetic patch aortoplasty [12], (iv) subclavian flap aortoplasty [13], and (v) balloon angioplasty [14]. All four surgical techniques are routinely performed, however there are no available clinical guidelines regarding the selection of the surgical technique. Hence, the selection process of the surgical process is mostly determined by individual preference of the surgeon.

The diagnosis and description of CoA are relatively simple, explaining and understanding the surgical treatment options without proper tools can be challenging. Since the leading complication after surgery is recoarctation [15], the proper education of clinicians regarding the surgical treatment options for CoA is crucial.

Kleszcz et al. [16] proposed a CoA simulation device to aid clinicians’ understanding of its surgical treatment options. This HOSTT was acquired by utilizing the following steps: (i) segmentation of a computed tomography angiography (CTA), (ii) virtual model preparation with CAD modeling, (iii) 3D printing of a rigid hollow model, and (iv) preparation of a solid wax core and silicone coating. The finished elastic phantom was mounted on a wooden board, and a polyvinyl pipe imitated the thoracic wall: A sponge was placed behind the aorta phantom, which was fixated with syringes and cable ties.

Therefore, this study aimed to propose a 3D printable HOSTT for the simulation of coarctation surgery and to investigate its effectiveness in the practical education of undergraduate medical students with no previous experience in this field.

## Materials and methods

### Study design

This practical coarctation surgical training involved undergraduate medical students in years two to six. All the participants signed written informed consent for both study participation and the publication of identifying information or images in an online open-access publication. This study was part of a more comprehensive investigation on applying 3D technologies in CoA treatment. The study protocol was approved by the Semmelweis University Regional and Institutional Committee of Science and Research Ethics (approval number: SE RKEB 202-1/2005). The study was conducted in full accordance with the declaration of Helsinki, as revised in 2013.

Participants were assigned into one of four groups depending on the surgical modality: Group A performed the technically least difficult end-to-end anastomosis (*n* = 5), Group B performed prosthetic patch aortoplasty (*n* = 5), Group C performed end-to-side anastomosis (*n* = 5), and group D performed the technically most challenging subclavian flap aortoplasty (*n* = 6). Participants were randomly assigned to each group using a random number generator (www.random.org). A survey form with directed questions was given to the participants before entering the theoretical and surgical training session and after completion. The model’s educative effectiveness was validated based on their responses. This study was performed at the 3D Center of Semmelweis University, Budapest, Hungary.

### HOSTT Fabrication

#### Virtual model acquisition

**Fig. 1:**
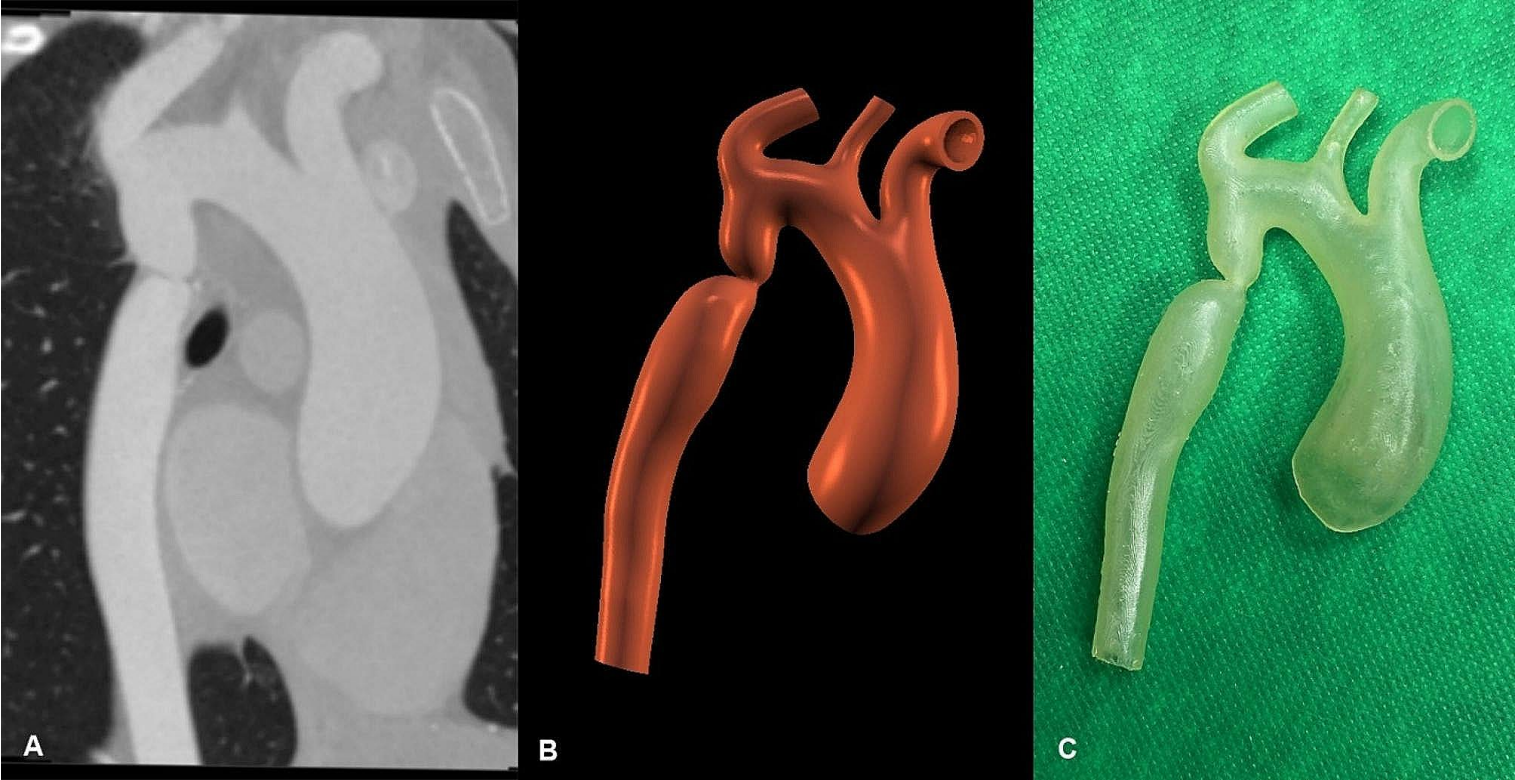
Coarctation morphology. **A**: Virtual 3D model of the coarctation. **B**: The processed 3D-printed model of the coarctation

The virtual 3D model of the aorta was acquired from a CTA scan of a three-year-old child diagnosed with congenital CoA. Digital Imaging and Communication in Medicine (DICOM) files were imported into an open-source radiographic image processing software (3D Slicer; www.slicer.org) [17] (Fig. 1A). The arch of the aorta, together with the supraaortic branches, were segmented using a global thresholding image segmentation method. Next, the “hollow” segment editor effect was used to generate a hollow segment with a uniform wall thickness of 0.6 mm. Then, the patient’s bony thorax was segmented using a global thresholding segmentation method. The cartilage between the ribs and sternum was reconstructed manually. After segmentation, the 3D surface representation of the aorta and the bony thorax was exported as a Standard Tessellation Language (STL) file [18] (Fig. 1B).

#### 3D printing

**Fig. 2:**
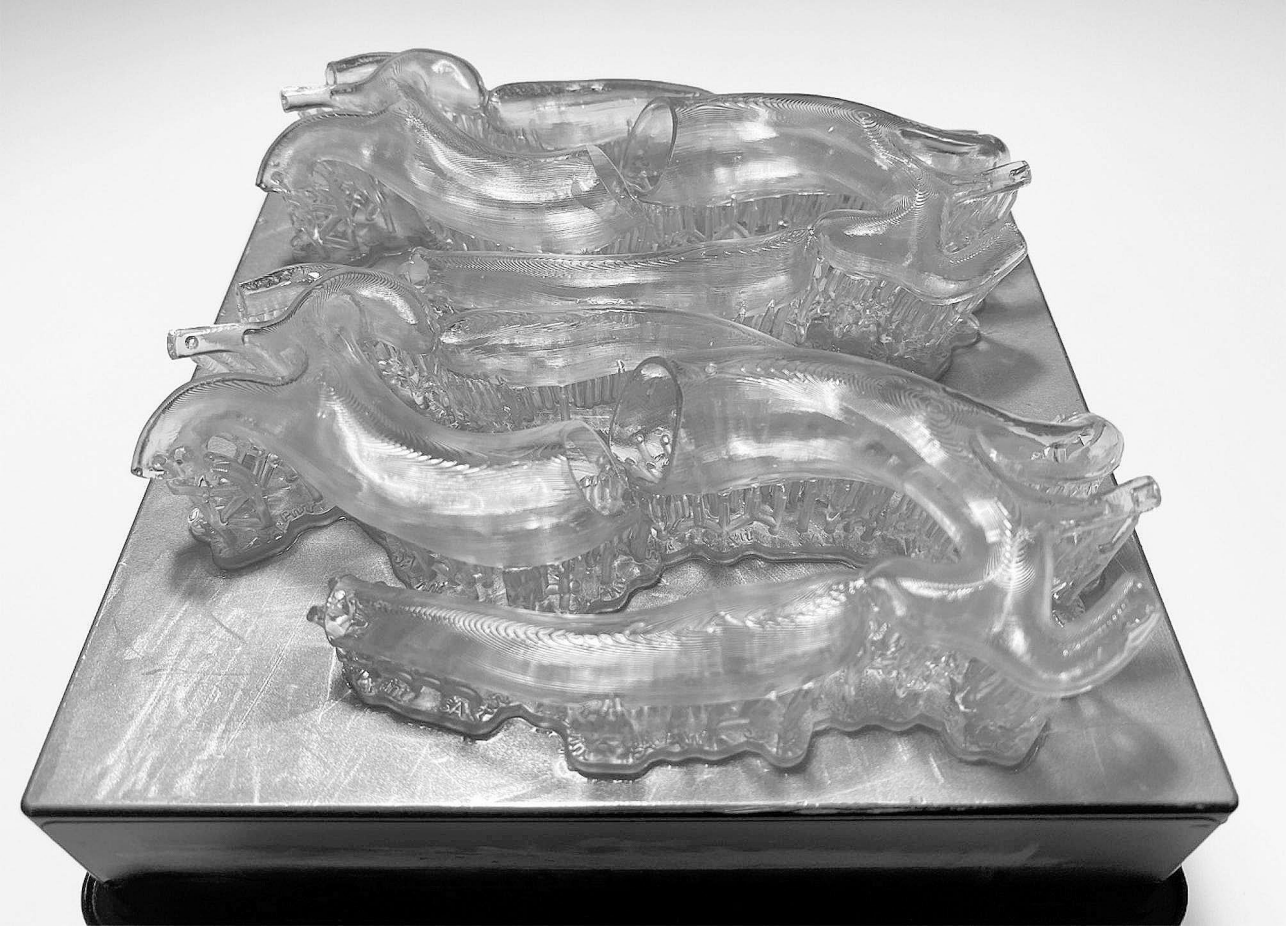
3D-printed models of the CoA manufactured from Elastic 50 A resin

The aorta phantom was fabricated with stereolithography (SLA) 3D printing technology using a Form 3B printer (Formlabs, Somerville, MA, USA) from an elastic resin (Elastic 50 A; Formlabs). Post-processing comprised a 20-minute wash cycle with isopropyl alcohol and a 60-minute curing process in a UV chamber (Form Cure; Formlabs). After curing, the material has ideal mechanical properties for surgical simulation, with a tensile strength of 3.23 MPa and a tear strength of 19.1 kN/m, according to the manufacturer’s technical data sheet (https://formlabs.com/store/materials/biomed-elastic-50a-resin/). The same aorta phantom was provided to all participants (Figs. 1C and 2).

**Fig. 3:**
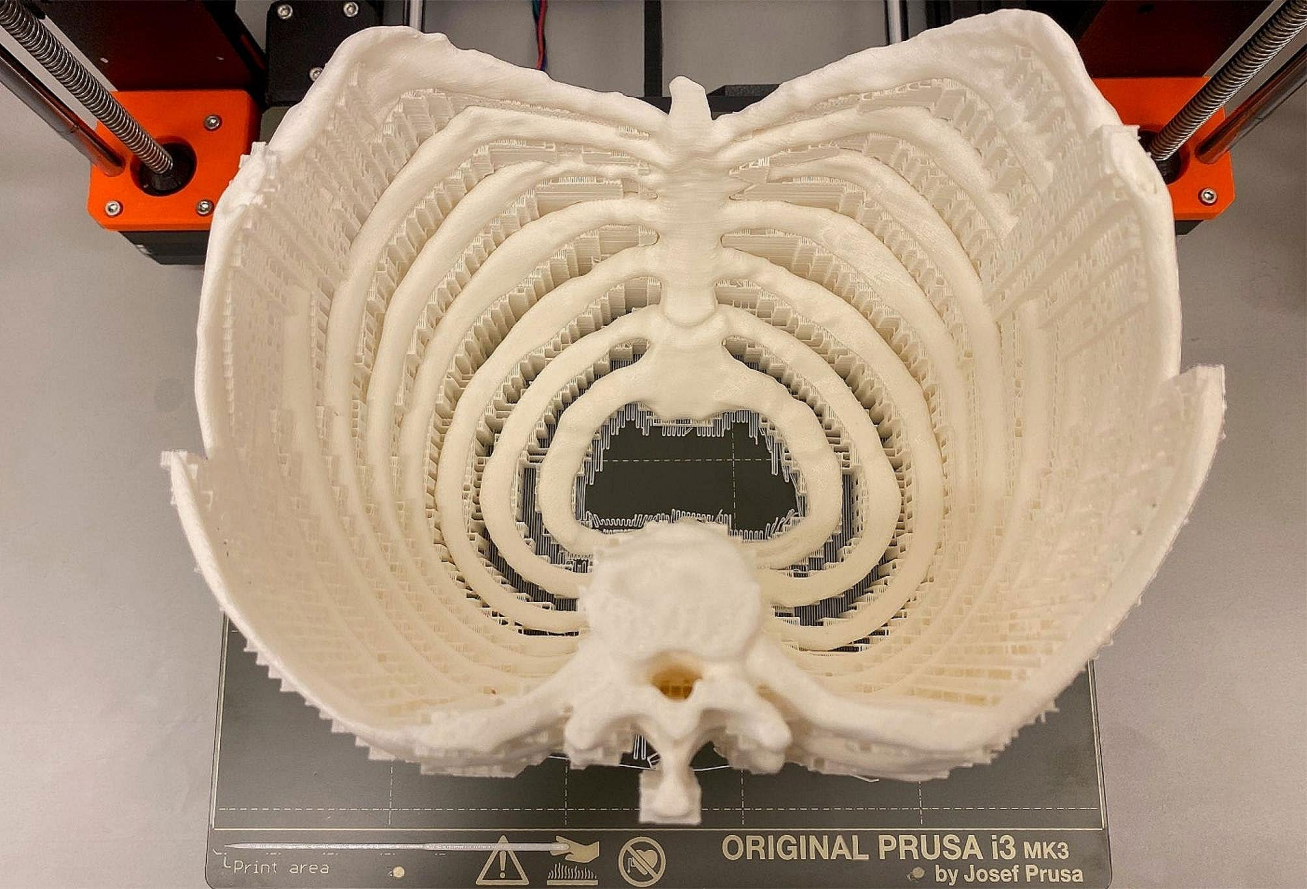
3D-printed model of the bony thorax on the print bed

The patient’s bony thorax was manufactured from a white-colored polylactic acid filament with fused filament fabrication (FFF) technology using a Prusa i3 MK3S + 3D printer (Prusa Research a.s., Prague, Czech Republic; Fig. 3).

#### HOSTT assembly

**Fig. 4:**
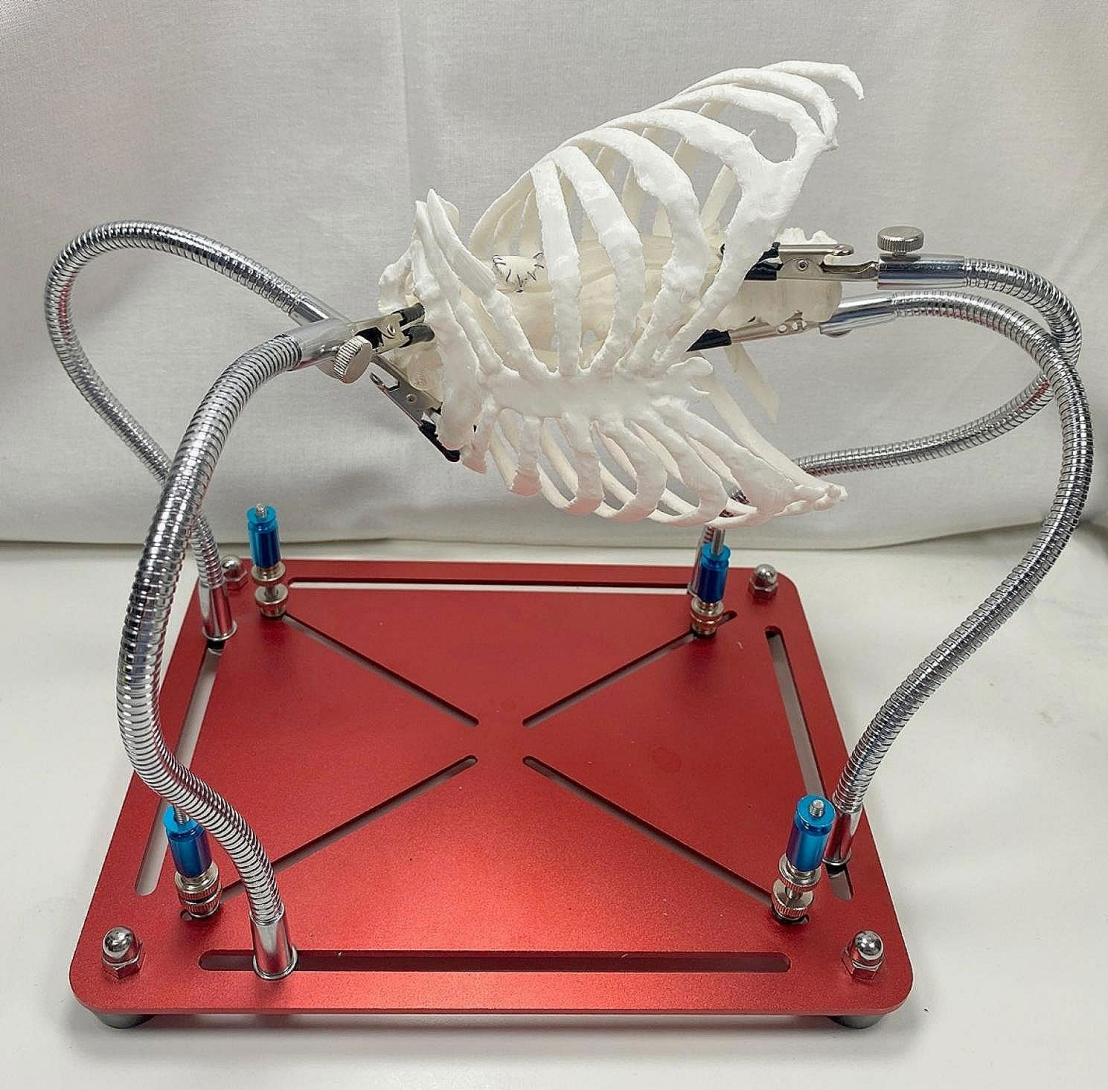
The 3D printed hands-on training tool (HOSTT) assembly for practical training of CoA surgery. The bony thorax was manufactured by FFF and the aorta phantom was manufactured from an elastic resin by SLA. Alligator clips hold the both 3D-printed models in place. The model of the bony thorax was modified after additive manufacturing, by heating at the 3rd intercostal space with a heat gun. Thus the space required for the surgery was created

The 3D-printed bony thorax was fixed on a plate using two alligator clips in the right lateral decubitus position, similarly to the real-life operating situation. The printed aorta with coarctation was placed in an approximate anatomical situation inside the bony thorax with two additional alligator clips. The lateral thoracotomy and retraction was imitated by heating and reforming the third intercostal space by 4 cm between the third and fourth 3D printed ribs (Fig. 4).

#### Surgical simulation

**Fig. 5:**
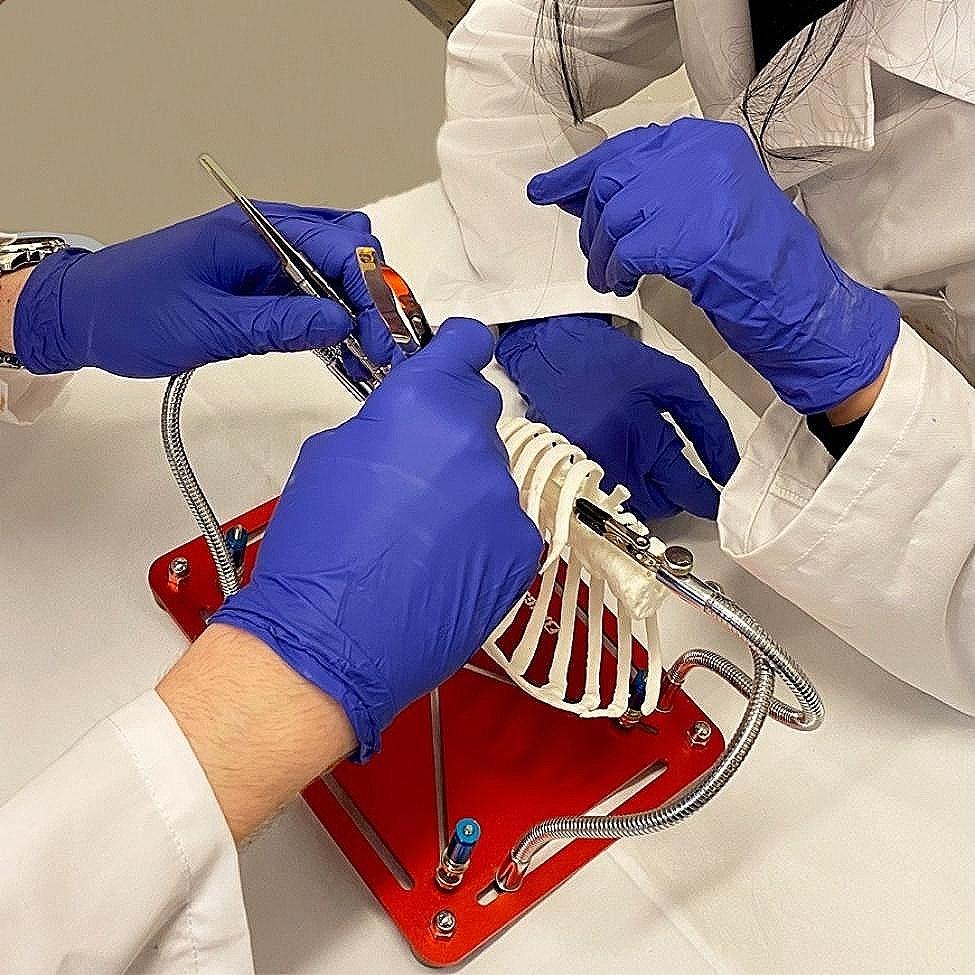
University students performing coarctation surgery with the 3D-printed HOSTT

Prior to randomization all participants attended a theoretical session and technical demonstration where the two experts described all four surgical modalities. Experts used images showing all four surgical techniques step-by-step and performed all four surgeries using the 3D HOSTT. Groups of four participants (one of each group) were assembled and performed surgeries one-by-one. So all students could witnessed the execution of all surgical modalities close by. Each participant received a 3D-printed aorta phantom showing coarctation. Following the education on all surgeries, each participant performed one of the four surgical procedures based on their study group: end-to-end anastomosis (Group A), extended end-to-end anastomosis (Group B), synthetic patch aortoplasty (Group C), and subclavian flap aortoplasty (Group D). During the training session, two trained professional cardiac surgeons (I.J.B. and I.H.) with at least 10 years of experience in the field supervised the participants’ work and provided support when necessary, but without interference (Fig. 5). The number of interactions with the tutors were recorded.

**Fig. 6:**
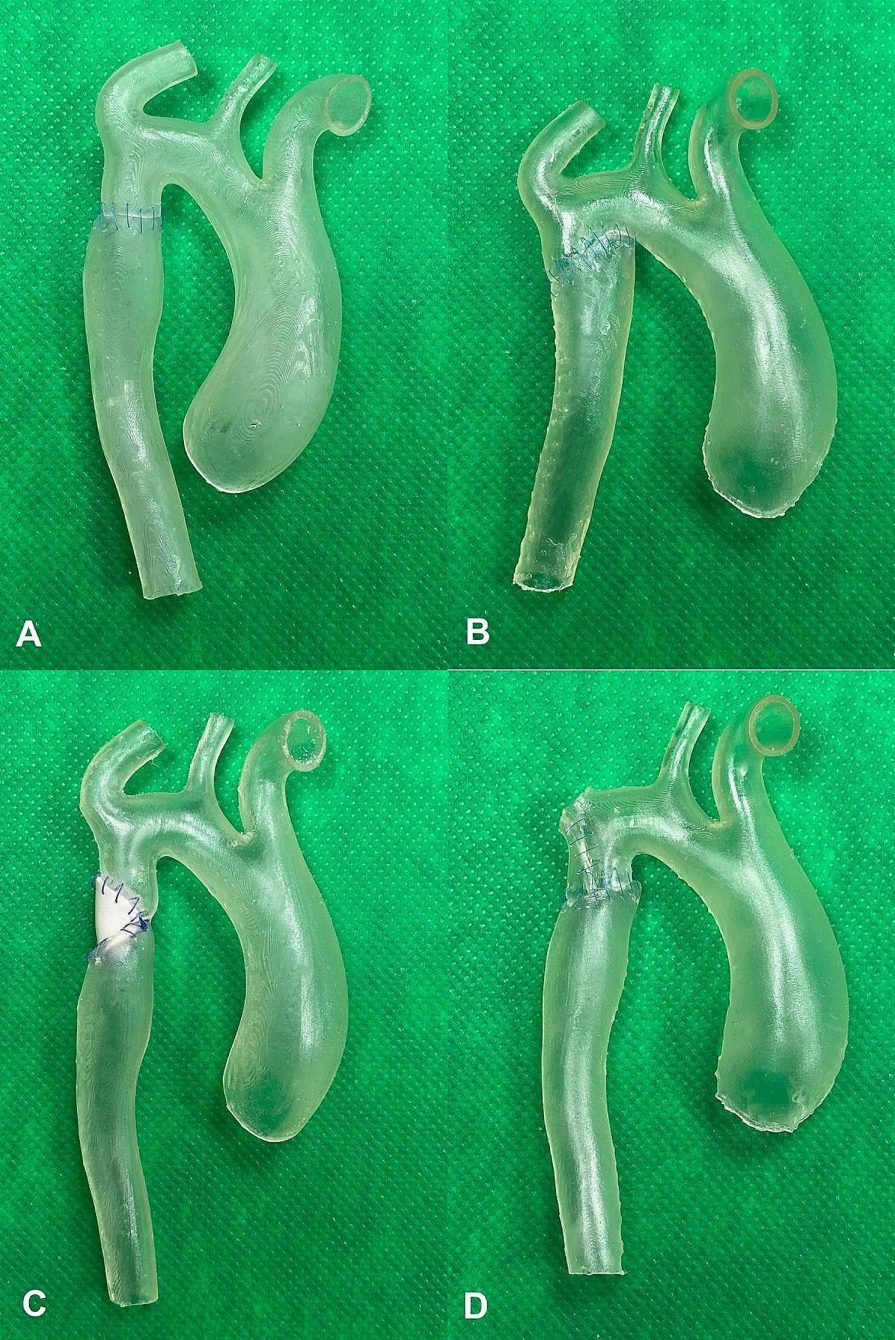
Results of different treatment options for the coarctation of the aorta simulated on elastic 3D-printed aorta phantoms. **A**: End-to-end anastomosis. **B**: End-to-side anastomosis. **C**: Prosthetic patch aortoplasty. **D**: Subclavian flap aortoplasty

All four surgical approaches were performed through a left thoracotomy at the third intercostal space:

- **End-to-end anastomosis**: the coarctation and ductal tissue is completely resected, and the proximal and distal aortic stumps are anastomosed [10] (Fig. 6A).

- **End-to-side anastomosis**: the aorta is cut under the narrowed segment, and the proximal part, the aortic arch is dissected along the lesser curvature, from the isthmus to the distal ascending aorta. Then, the distal end is anastomosed to this incision [11] (Fig. 6B).
- **Prosthetic patch aortoplasty**: a longitudinal incision opens the aorta anteriorly, through the coarcted segment. A polyethylene terephthalate (Dacron) patch is stitched into the aortotomy using continuous suture [12] (Fig. 6C).
- **Subclavian flap aortoplasty**: an incision is made through the coarctation, the isthmus and the proximal segment of the left subclavian artery, which is then rotated downwards as a flap and sutured to the two sides of the aortotomy. (Fig. 6D).

Outcome Variables—Participant Feedback.

The participants’ comprehension of the clinical problem of CoA and its surgical treatment was evaluated by asking the same questions before and after the theoretical explanation, presentation and training session. Participants rated their understanding of each question on a seven-point Likert scale from 1 to 7 [19]. The following directed questions were asked:

1. How well do you understand the CoA disease and its treatment options? **(Primary question)**
2. How well do you understand the localization of coarctation in the aorta?
3. How well do you understand the risks and complications of coarctation surgery?
4. How well do you understand the end-to-end anastomosis technique?
5. How well do you understand the end-to-side anastomosis technique?
6. How well do you understand the prosthetic patch aortoplasty technique?
7. How well do you understand the subclavian flap technique?

Participant responses were collected via Google Forms (Google, Mountain View, CA, USA).

In addition to investigating participant feedback, necessary interventions by the two professional cardiac surgeons were counted.

### Statistical analysis

Data are expressed as mean ± standard deviation. Descriptive statistics were used for all ratings provided by the participants. The normality of the examined variables was assessed using the Shapiro–Wilk test. The homogeneity of the variances between the groups was assessed using Levene’s test. Data was found to be non-normally distributed and to have inhomogeneous variance. Therefore, non-parametric statistics were used to assess the statistical significance of differences in ratings corresponding to the answers given at different time points. The Wilcoxon matched-pairs signed-rank test was used, considering a significance level of 0.05. Statistical analyses were conducted in SPSS Statistics 21 (IBM, Armonk, NY, USA).

## Results

### Participant demographics

This study included 21 medical students from Semmelweis University, of which 10 were male and 11 were female, with a mean age of 23.19 ± 1.25 years. Regarding the educational year, one participant was in their second year, six were in their third year, six were in their fourth year, seven were in their fifth year, and one was in his/her sixth year. Study demographic data are summarized in Table 1.

**Table 1:** Participants’ demographic data

Participant	Age	Sex	Educational year	Performed surgery	Number of interactions with the tutor
1	23	M^1^	4th	SUB^3^	1
2	21	F^2^	3rd	E2S^4^	0
3	22	F	3rd	SUB	1
4	25	F	5th	SUB	2
5	24	M	5th	SUB	1
6	22	F	3rd	PATCH^5^	1
7	23	F	5th	SUB	0
8	24	M	4th	E2E^6^	0
9	22	M	4th	E2S	2
10	21	M	2nd	E2S	0
11	22	F	3rd	SUB	2
12	24	M	5th	PATCH	1
13	22	M	4th	PATCH	1
14	24	F	5th	PATCH	1
15	24	M	5th	E2S	1
16	25	F	6th	PATCH	0
17	23	M	3rd	E2S	1
18	23	M	3rd	E2E	1
19	24	F	4th	E2E	0
20	24	F	4th	E2E	0
21	25	F	5th	E2E	0

Key: ^1^male, ^2^female, ^3^subclavian flap, ^4^end-to-side anastomosis, ^5^prosthetic patch aortoplasty, ^6^end-to-end anastomosis

### Participant feedback—primary outcome

This study’s primary aim was to evaluate participants’ understanding of CoA disease using the 3D-printed HOSTTs (primary survey question: “How well do you understand the CoA disease and its treatment options?”). Participants’ average rating of their understanding of CoA diseases before practical training was 4.62 ± 1.07 on a seven-point Likert scale. After training, their average rating increased to 6.19 ± 1.08. The Wilcoxon matched-pairs signed-rank test indicated that pre- and post-training ratings differed significantly (*p* < 0.05).

#### Participant feedback—secondary outcome

Secondary aim was to evaluate participants’ understanding of other relevant aspects of CoA and its treatment. A statistical evaluation of participants’ responses indicated that they had significantly ( *p* < 0.05) greater knowledge of coarctation localization after practical training (*p* < 0.05). Participants’ average ratings before and after the practical training were 3.19 ± 1.06 and 6.48 ± 0.51, respectively. However, their pre- and post-training ratings for risks and complications of CoA surgery did not differ significantly (*p* = 0.26):5.86 ± 1.06 and 6.00 ± 1.00, respectively.

Regarding surgical treatment options, participants’ understanding of three of the four surgical modalities differed significantly pre- and post-training. However, their understanding of patch aortoplasty did not differ significantly pre- and post-training (*p* = 0.32). Participants’ average pre-training ratings for their understanding of end-to-end anastomosis, end-to-side anastomosis, prosthetic patch aortoplasty, and the subclavian flap technique were 4.71 ± 1.49, 5.33 ± 1.11, 6.57 ± 0.51, and 2.86 ± 1.39, respectively. In contrast, their average post-training ratings were 6.48 ± 0.51, 6.67 ± 0.48, 6.62 ± 0.50, and 6.05 ± 0.97, respectively. The data is summarized in Table 2.

**Table 2:** Participants’ level of understanding on a seven-point Likert scale

Question		Before	After	p value^3^
How well do you understand the CoA^1^ disease?	Mean ± St. dev.^2^	4.62 ± 1.07	6.19 ± 1.08	< 0.05
	Median	5.00	7.00	
How well do you understand the localization of CoA?	Mean ± St. dev.	3.19 ± 1.60	6.48 ± 0.51	< 0.05
	Median	3.00	6.00	
How well do you understand the risks and complications of coarctation surgery?	Mean ± St. dev.	5.86 ± 1.06	6.00 ± 1.00	0.260
	Median	6.00	6.00	
How well do you understand the end-to-end anastomosis technique?	Mean ± St. dev.	4.71 ± 1.49	6.48 ± 0.51	0.001
	Median	4.00	6.00	
How well do you understand the end-to-side anastomosis technique?	Mean ± St. dev.	5.33 ± 1.11	6.67 ± 0.48	< 0.05
	Median	5.00	7.00	
How well do you understand the prosthetic patch aortoplasty technique?	Mean ± St. dev.	6.57 ± 0.51	6.62 ± 0.50	0.320
	Median	7.00	7.00	
How well do you understand the subclavian flap technique?	Mean ± St. dev.	2.86 ± 1.39	6.05 ± 0.97	0.05
	Median	3.00	6.00	

Key: ^1^Coarctation of the aorta, ^2^standard deviation, ^3^Wilcoxon matched-pairs signed-rank test

#### Execution of surgical simulation

After a thorough explanation of each treatment modality, all participants performed one of four surgical techniques using the 3D-printed phantoms. During the session, minimal oral support was necessary by the appointed professionals. Group C required the most interactions (*n* = 7), and Group A required the fewest (*n* = 1). Groups B and D needed a total of four interventions each by the professionals.

## Discussion

This study used an HOSTT with 3D-printed aortic phantoms for practical training in CoA treatment. The primary aim of the study was to investigate the effectiveness of 3D-printed phantoms in the practical training of medical students. To validate the usability of the 3D-printed HOSTT in the current examination, medical students with no previous experience in CoA treatment have performed one of four treatment options [20]. Participants’ knowledge and understanding of CoA and its treatment were assessed based on their responses to seven questions before and after the training session.

Participants’ understanding increased significantly in all but two investigated aspects of CoA: (i) risk factors and complications of coarctation surgery and (ii) the prosthetic patch aortoplasty surgical treatment option. This result is also supported by the number of necessary interventions by the cardiac surgeons, which was the lowest in the group performing end-to-end anastomosis. Despite its limited number of participants, this study demonstrated that the HOSTT can be used as an effective practical training tool for surgical CoA treatment. Participants with no prior experience in this area could successfully perform even the most challenging treatment modality (subclavian flap technique) with a relatively low number of professional interactions. The patient-specific HOSTT used in this study could also be used in clinical practice during the presurgical treatment phase by surgeons to better understand the defect morphology. The clinical implementation of this setup could reduce the rate of unforeseeable intraoperative complications, achieving better surgical results [21, 22].

Compared to the method utilized by Kleszcz [16], the construction of the 3D printed HOSTT used in this study was relatively simple. The assembly of our model did not require layer-by-layer silicone molding—which could be a very lengthy process - to acquire the aorta phantom. Although, our process would require the use of a relatively expesive 3D printable elastic material.

With the ability to 3D print elastic materials that can realistically replicate vessels, cumbersome silicone molding processes can be avoided. All the surgical approaches were performed through a left thoracotomy at the third intercostal space. To simulate the entry point and access to the surgical area, the HOSTT also contained a 3D-printed model of the patient’s bony thorax which made the treatment simulation even more realistic. Methods using virtual and augmented reality are becoming more and more common in the training of professionals and during preoperative planning. Virtual reality has the advantages of being able to enlarge objects, toggle visibility, and set opacity levels for components in the surgical training model [23, 24]. However, its evident shortcoming is the lack of realistic haptic feedback that physical models provide.

This study had some notable limitations, the most prominent of which was the inability to replicate exact surgical conditions. Developing a 3D printable soft tissue analog material would allow fully simulated surgical access. Another drawback of the HOSTT used is that, while the 3D printable elastic material can simulate CoA surgery, its physical properties and handling do not accurately resemble actual human tissues and intraoperative conditions. These issues must be addressed by developing materials to construct an even more realistic HOSTT. Another limitation of this study was its relatively limited number of participants. Nevertheless, the effectiveness of the HOSTT could still be demonstrated.

## Conclusions

Within this study’s limitations, the HOSTT manufactured using SLA and FFF 3D printing technologies was shown to be suitable for the practical training of surgical treatment methods for CoA. Even medical students with no prior experience could perform all surgical techniques with relatively low number of interventions by professionals. While only a limited number of students participated in this study, based on their feedback, the HOSTT was effective in increasing their knowledge of CoA and its treatment.

## Data Availability

The datasets used and/or analyzed during this study are available from the corresponding author upon reasonable request.

## Abbreviations

CoA: Coarctation of the aorta
SBM: Synthetic bench model
CAD: Computer-aided design
CTA: Computed tomography angiogarphy
DICOM: Digital Imaging and Communication in Medicine
STL: Standard tessellation language
SLA: Stereolithography
FFF: Fused filament fabrication
